# HEV-associated dendritic cells are observed in metastatic tumor-draining lymph nodes of cutaneous melanoma patients with longer distant metastasis-free survival after adjuvant immunotherapy

**DOI:** 10.3389/fimmu.2023.1231734

**Published:** 2023-08-25

**Authors:** Alicia Inés Bravo, Mariana Aris, Marylou Panouillot, Martina Porto, Marie-Caroline Dieu-Nosjean, Jean-Luc Teillaud, María Marcela Barrio, José Mordoh

**Affiliations:** ^1^Laboratorio de Cancerología, Fundación Instituto Leloir, Ciudad Autónoma de Buenos Aires (CABA), Argentina; ^2^Unidad de Inmunopatología, Hospital HIGA Eva Perón, Provincia de Buenos Aires, Buenos Aires, Argentina; ^3^Centro de Investigaciones Oncológicas, Fundación Cáncer (FUCA), Ciudad Autónoma de Buenos Aires (CABA), Argentina; ^4^Sorbonne University, Faculty of Medicine, UMRS 1135, Laboratory “Immune Microenvironment and Immunotherapy”, Centre d’Immunologie et des Maladies Infectieuses (CIMI), Paris, France; ^5^Inserm U.1135, Laboratory “Immune Microenvironment and Immunotherapy”, Centre d’Immunologie et des Maladies Infectieuses (CIMI), Paris, France; ^6^Laboratory “Immune Microenvironment and Immunotherapy”, Centre d’Immunologie et des Maladies Infectieuses (CIMI), Paris, France

**Keywords:** cutaneous melanoma, dendritic cells, distant metastasis-free survival, high endothelial venules, metastatic tumor-draining lymph nodes

## Abstract

**Introduction:**

Tissue biomarkers that aid in identifying cutaneous melanoma (CM) patients who will benefit from adjuvant immunotherapy are of crucial interest. Metastatic tumor-draining lymph nodes (mTDLN) are the first encounter site between the metastatic CM cells and an organized immune structure. Therefore, their study may reveal mechanisms that could influence patients´ outcomes.

**Methods:**

Twenty-nine stage-III CM patients enrolled in clinical trials to study the vaccine VACCIMEL were included in this retrospective study. After radical mTDLN dissection, patients were treated with VACCIMEL (n=22) or IFNα-2b (n=6), unless rapid progression (n=1). Distant Metastasis-Free Survival (DMFS) was selected as an end-point. Two cohorts of patients were selected: one with a good outcome (GO) (n=17; median DMFS 130.0 months), and another with a bad outcome (BO) (n=12; median DMFS 8.5 months). We analyzed by immunohistochemistry and immunofluorescence the expression of relevant biomarkers to tumor-cell biology and immune cells and structures in mTDLN, both in the tumor and peritumoral areas.

**Results:**

In BO patients, highly replicating Ki-67^+^ tumor cells, low tumor HLA-I expression and abundant FoxP3^+^ lymphocytes were found (*p=0.037; p=0.056* and *p=0.021*). In GO patients, the most favorable biomarkers for prolonged DMFS were the abundance of peri- and intra-tumoral CD11c^+^ cells (*p=0.0002* and *p=0.001*), peri-tumoral DC-LAMP^+^ dendritic cells (DCs) (*p=0.001*), and PNAd^+^ High Endothelial Venules (HEVs) (*p=0.004*). Most strikingly, we describe in GO patients a peculiar, heterogeneous structure that we named FAPS (Favoring Antigen-Presenting Structure), a triad composed of DC, HEV and CD62L^+^ naïve lymphocytes, whose postulated role would be to favor tumor antigen (Ag) priming of incoming naïve lymphocytes. We also found in GO patients a preferential tumor infiltration of CD8^+^ and CD20^+^ lymphocytes (*p=0.004* and *p=0.027*), as well as peritumoral CD20^+^ aggregates, with no CD21^+^ follicular dendritic cells detected (*p=0.023*). Heterogeneous infiltration with CD64^+^CD68^-^CD163^-^, CD64^+^CD68^+^CD163^-^ and CD64^+^CD68^+^CD163^+^ macrophages were observed in both cohorts.

**Discussion:**

The analysis of mTDLN in GO and BO patients revealed marked differences. This work highlights the importance of analyzing resected mTDLN from CM patients and suggests a correlation between tumor and immune characteristics that may be associated with a spontaneous or vaccine-induced long DMFS. These results should be confirmed in prospective studies.

## Introduction

Left to itself, cutaneous melanoma (CM) is a fast-growing, devastating disease. There is consensus that the outcome of CM is a balance between the speed of tumor growth, its invasiveness and the efficiency of the immune system (IS) to raise defenses against it. Fortunately, the finding that 50% of CM patients have a mutation in the BRAF oncogene led to the synthesis of inhibitors targeting this mutation. The use of inhibitors against BRAF^V600^ and MEK produced great advances in CM treatment (1). However, the treatment with inhibitors of the MAP Kinase pathway usually generates resistance (2). Another remarkable advance was the discovery of the immunosuppressive axes CTLA-4/CD28 (3) and PD-1/PD-L1 (4), which led to the synthesis of monoclonal antibodies (mAbs) against such pathways (Immune Checkpoint Inhibitors, ICI). The use of such mAbs, especially anti-PD-1, has led to major advances in the therapy of various tumors, among them CM. However, only a part of the CM population treated with ICI, either in metastatic disease or adjuvancy, responds to treatment (5, 6). Therefore, it is important to investigate the reasons for such disparate evolution to immunotherapy (IT). Metastatic tumor-draining lymph nodes (mTDLN) are the most common encounter site between the metastatic CM cells and an organized immune structure. Therefore, their study may reveal interaction mechanisms that could influence patients´ outcomes. Several attempts have been made to establish prognostic factors to differentiate progressor from non-progressor CM patients. Sivendran et al. analyzed mRNA from primary tumors from stages II-III CM patients and established a 53–gene panel that predicted spontaneously progressing from non-progressing patients, CD2 being the most expressed gene in the panel (7). Erdag et al. analyzed immune infiltrates in 183 metastases from 147 CM patients, 83 of whom (44%) were in lymph nodes (LN) (8). The authors defined histological immunotypes A (no immune infiltrate), B (immune infiltrate limited to surroundings of blood vessels) and C (immune infiltrate within tumors), which correlated with disease progression (A>B>C). Bogunovic et al. analyzed by gene chip technology the mRNA expression in LN from 38 CM patients and found 266 genes related to patients´ survival; the genes that positively correlated with survival were associated with the immune response and those that negatively correlated were involved in cell replication (9). More recently, Yaddanapudi et al. performed a single-cell analysis of immune cells in sentinel LN from 41 stage III CM patients and concluded that an immune-tolerant microenvironment was predominant in mTDLN (10).

Under the premise that challenging the IS with multiple antigens (Ags) would enhance the immune responsive repertoire of T-cell clones, we have developed the therapeutic vaccine VACCIMEL, composed of allogeneic irradiated CM cells adjuvanted with BCG and GM-CSF. VACCIMEL has been studied in CM patients in Phase I (11, 12) and Phase II studies (13, 14). The recently updated CASVAC 0401 Phase II study was an open-label, adjuvant, randomized study on stages IIB, IIC and III CM patients, in which VACCIMEL was compared in a 2:1 ratio to medium-dose IFN-α2b (15). In this study, after a minimum and maximum follow-up of 91 and 150 months, respectively, DMFS was significantly longer in vaccinated patients than in IFN-α2b treated patients with median DMFS of 96 months and 13 months (*p=0.035*). In these studies, some patients had quite a long DMFS, while others recurred rapidly. These results strongly suggest that some patients´ intrinsic characteristics, both tumor- and/or immune-related, present even before treatment, may influence the course of the disease. To investigate this possibility, two cohorts of stage III CM patients with extremely different outcomes after adjuvant IT were selected: a Good Outcome (GO) cohort with long DMFS and a Bad Outcome (BO) cohort with short DMFS. We analyzed mTDLN to understand in this environment which are the characteristics of tumor and immune cells that may influence the course of the disease.

## Materials and methods

### Patients

To perform this retrospective, exploratory study, 29 stage III CM patients were selected from previous phase I (11, 12) and phase II clinical studies (13–15) spanning between 1992 and 2016. All the patients had undergone radical lymphadenectomy. We analyzed mTDLN biopsies obtained after surgery and before any subsequent treatment. Two cohorts of patients with widely different outcomes were selected: Good Outcome (GO) patients (n=17) and Bad Outcome (BO) patients (n=12) by setting an upper limit for DMFS of 20 months for the BO cohort and a lower limit for DMFS of 50 months for the GO cohort after immunotherapy. Twenty-nine patients fulfilled four conditions: i) being at stage III (AJCC 8th edition) of their disease (16); ii) having an updated follow-up; iii) having good quality biopsies; iv) having been treated after lymphadenectomy with VACCIMEL or IFN-α2b. The patients’ characteristics, the treatments received and DMFS are detailed in **Supplementary Table 1**. The median age of GO patients was 44.0 years and 7/17 were females (41%). As it refers to BO patients, the median age was 43.0 years and 5/12 were females (41.7%). Thus, the median ages and female:male ratios were similar in GO and BO cohorts.

As it refers to the characteristics of the primary tumors, differences were noticed. In the GO cohort, in 5/17 (29.4%) of the patients, the primary lesion could not be detected, whereas in the BO cohort only 1/12 (8.3%) patient had its primary tumor undetectable. The higher percentage of unknown primary tumors in the GO cohort is in accordance with the results reported by Tarhini et al., who reported a better prognosis in CM patients with unknown primary tumor detected (17). As it refers to the Breslow index, the median index for the GO patients was 2.1 mm, whereas the median Breslow index for the BO cohort was 5.0 mm (Wilcoxon test, p=0.011). As to the ulceration of primary tumors, it was less frequent in GO patients: 5/12 (41.7%) than in BO patients: 8/10 (80%). Thus, as predictable, the BO cohort had worse prognostic features in their primary tumors than the GO population.

### Immunohistochemistry assays

Immunohistochemistry assays were performed as previously described (16). Primary mAbs: CD4 (clone SP35, 1:200, Abcam); CD8 (clone C8/144B, 1:200, Dako); CD20 (clone L26, 1:200, Dako); FoxP3 (clone 236AE7, 1:100, Abcam); CD68 (clone PG-M1, 1:200, Dako); CD163 (clone 10D6, 1:25, Invitrogen); CD11c (clone EP 1347Y, 1:200, Abcam); DC-LAMP (clone 1010E1.01, 1:100, Dendritics); Ki-67 (clone MB1, 1:150, Dako); HLA (clone EMR8-5, 1:350, Abcam); PD-1 (clone NAT 105, 1:200, Abcam); PNAd (clone MECA-79, 1:100, BD Pharmingen). The histological analysis of the metastatic tumor-draining lymph node (mTDLN) was done by an experienced pathologist in a blinded way; i.e. without knowing to which patient or cohort the slides belonged. Analysis was performed with an Olympus BX40 microscope using the DP2-BSW software, and counting was done with the Image J software. For each immunohistochemical marker, the slide was examined at 200X magnification in its totality, focusing on the tumor and peritumoral areas. For counting at 400X magnification, a field was chosen that represented in adequate proportions the different fields containing high, medium or low numbers of the analyzed marker. The counting was not performed in parasinusoidal or perivenular areas, only tumoral and peritumoral areas were examined. The peritumoral areas were defined as the area spanning from the invasive tumor border until two 400X fields apart (750 µm). HEV were counted in one field at 100x magnification, both in intra- and peritumoral areas.

For Ki-67 determination; only tumor cells according to their morphology were counted. For intratumoral immune cells, after counting, a ratio was established between the number of immune cells and the total number of tumor cells present in the selected field. The number of total tumor cells in the selected field was calculated by dividing the tumor surface area by the surface area of an average-sized tumor cell. Given that tumor cell sizes vary among patients, the cell areas were calculated individually for each chosen field with the DP2-BSW program. For HLA counting, only when the cell membranes were stained, the cells were deemed positive. HLA-I positive and negative cells were counted. High Endothelial Venules (HEVs) were detected as PNAd^+^ structures and counted in representative 100X fields, both intratumorally and at the peritumoral border. Peri-tumoral CD20^+^ aggregates were counted similarly.

### Multiplex immunofluorescence assays

As a result of IHC scoring analysis, selected biopsies from GO and BO patients were further analyzed by multiplex immunofluorescence. Multiplex immunofluorescence assays were performed and analyzed as previously described (18). Primary mAbs: CD11c (clone EP 1347Y, 1:200, Abcam); MART-1 conjugated to AF647 [IgG1, κ, clone 2A9 (19)]; TYR conjugated to AF647 (clone T311, Santa Cruz Biotechnology); CD62L mAb conjugated to AlexaFluor-647 (clone DREG56, 1:50, IgG1 κ, Santa Cruz Biotechnology). Secondary abs: Cy2 goat-anti-rat IgG [clone 712-225-153, Jackson ImmunoResearch (JIR)] and Cy3 F(ab’)_2_ donkey anti-rabbit IgG (clone 711-166-152, JIR).

Some multiplex immunofluorescence assays were amplified using anti-mouse IgG1-HRP (JIR) and the tyramide signal amplification system (Interchim). CD163 antibody (clone 10D6, 1:25, mouse IgG1, κ), CD64 (clone OTI3D3, 6.7µg/ml, mouse IgG1, κ); CD68 (clone KP1, 3.7µg/ml, Dako); CD20 (clone L26, 0,57µg/ml, mouse IgG2a, κ) (Dako) GP100 (clone HMB45, 1:50, mouse IgG1, κ) DC-LAMP (clone 1010E1.01, 1:100, rat IgG2a, κ) (Eurobio Scientific) anti-CD3 (rabbit polyclonal IgG, 8µg/ml, Dako); anti-CD21 (clone 1F8, 6.67µg/ml, mouse IgG1κ, Dako).

Imaging of the slides was performed using a Zeiss Axio Observer Z1 at 385nm, 430nm, 475nm, 511nm, 555nm, 590nm and 630nm with the corresponding filters and the Zen software (Zeiss).

### Statistical analyses

Data were analyzed with GraphPad Prism v10.0 for statistical analyses. Non-parametric Mann-Whitney test was performed to compare IHC score values from GO and BO cohorts; a p-value ≤ 0.05 was considered statistically significant.

## Results

### Immunohistochemical analysis of tumor cells, immune infiltrates and supra-cellular structures

**Figure 1:**
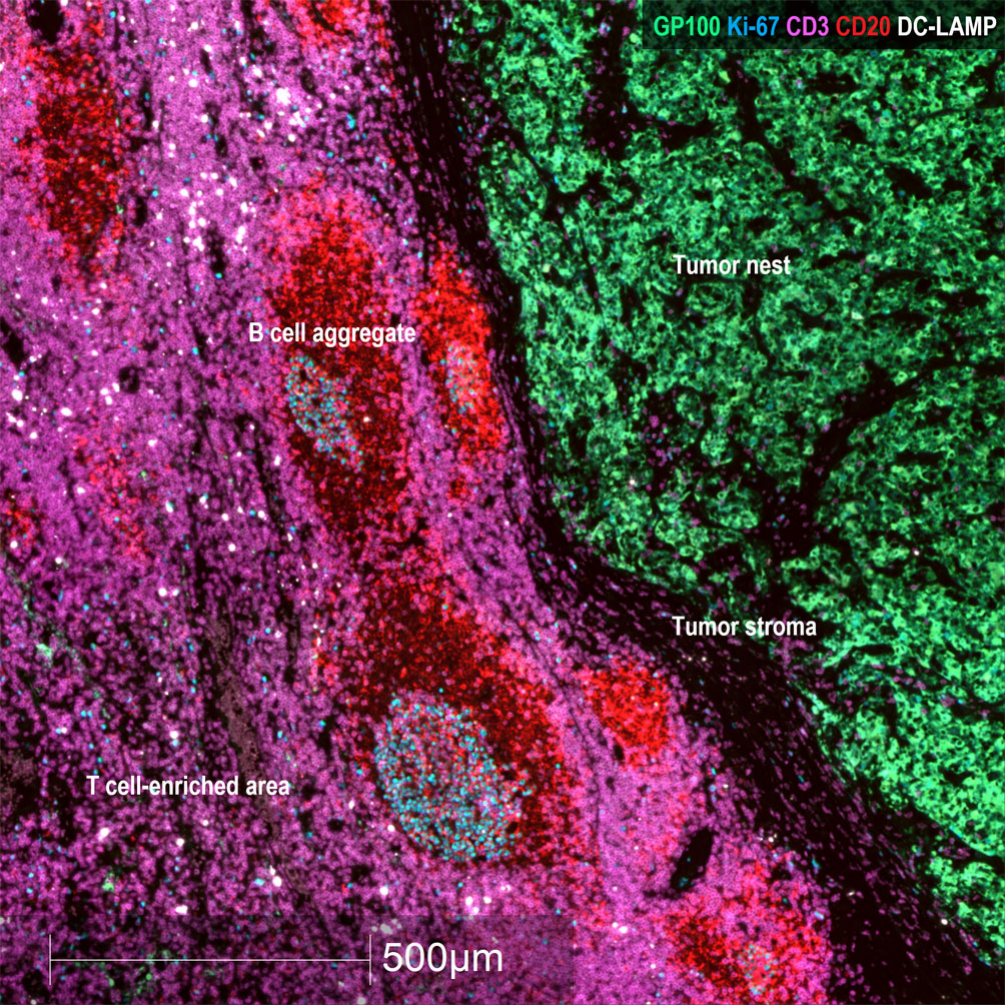
Representative areas in mTDLN biopsies. Multiplex immunofluorescence staining with anti-GP100 (green), anti-Ki-67 (blue), anti-CD3 (purple), anti-CD20 (red) and anti-DC-LAMP (white) antibodies was performed as described under Methods. Representative areas of the tumor (containing tumor nest and tumor stroma) and peritumoral area (containing B cell aggregates and T cell-enriched area) (patient#2) are shown. Scale bar: 500μm.

We analyzed two “endpoint” biomarkers of different essential tumor pathways: Ki-67 as a measure of the proliferation rate of the metastatic tumor and HLA-I expression as an indicator of the tumor susceptibility to cytotoxic lymphocytes. We also analyzed mTDLN tumor infiltration by diverse immune cell populations: Antigen-Presenting Cells (APC) (CD11c, DC-LAMP); Tumor-Associated Macrophage (TAM) subpopulations (CD68 and CD163); lymphocyte subpopulations (CD4, FoxP3, CD8 and CD20); and lymphoid structures such as PNAd^+^ High Endothelial Venules (HEVs) and CD20^+^ aggregates. It should be emphasized that the analyzed mTDLN were extracted before any treatment was administered. Thus, the expression of these biomarkers could shed light on the individual ability of the patients´ immune cells to interact with the tumor. To better understand the mTDLN areas in which cells or structures were counted, see **Figure 1**. These areas were defined as the tumor (containing tumor nest and tumor stroma) and the peritumoral area (containing B cell aggregates and T cell-enriched areas).

**Figure 2:**
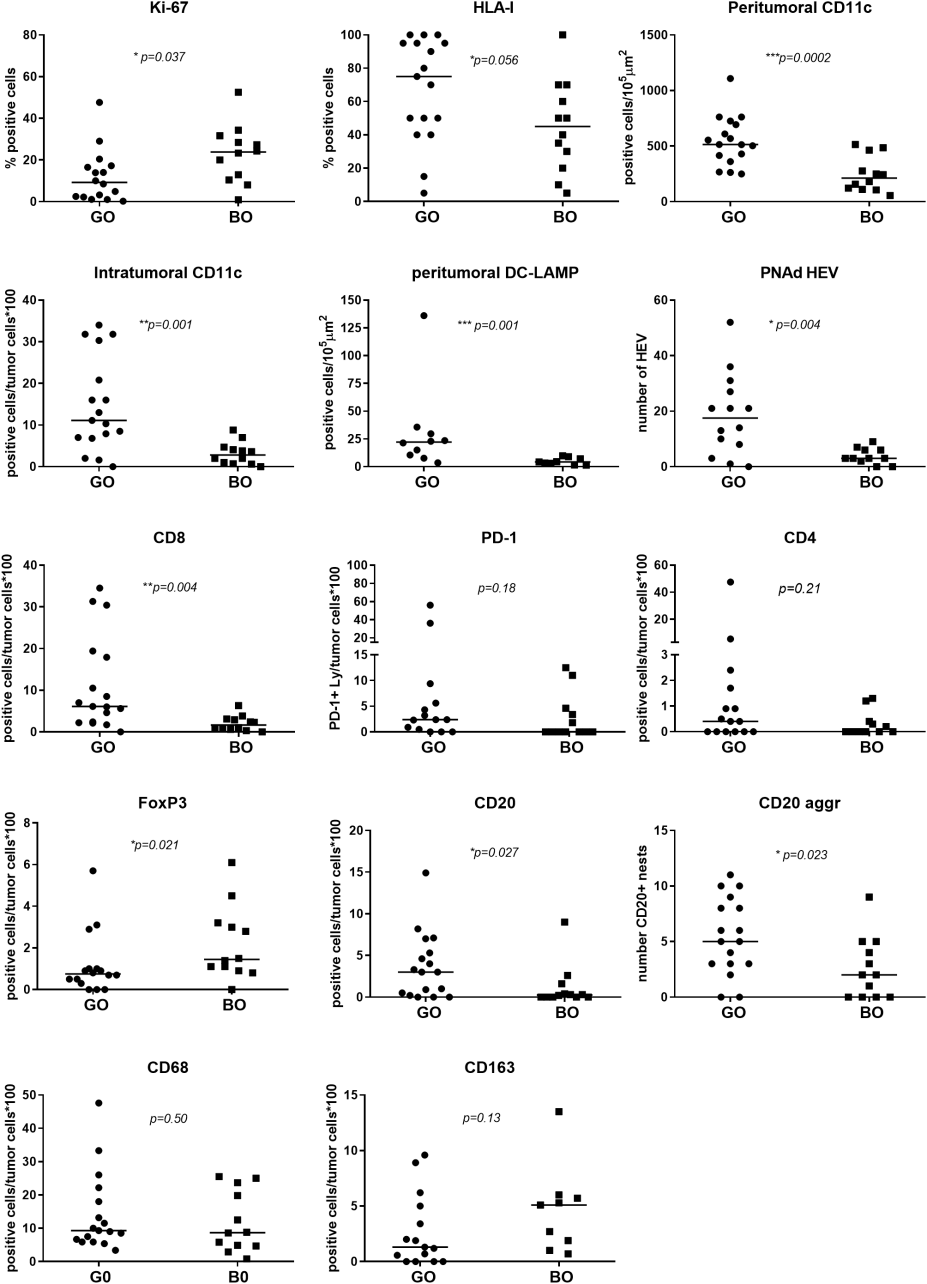
Comparison of tumor and immune cell biomarkers expression between GO and BO patients. IHC quantification is described in **Table 1**. A Mann-Whitney test was performed to compare GO and BO cohorts; a p-value ≤ 0.05 was considered statistically significant.

Representative examples of tumors with high and low numbers of HLA-I^+^ tumor cells, CD11c^+^ dendritic cells and CD8^+^ lymphocytes are shown in **Supplementary Figure 1**. After counting in representative fields as described under Methods, the results are detailed in **Table 1** and **Figure 2**.

**Table 1:** Quantification of biomarkers by IHC.

°Patient	Ki67^a^	HLA-I^a^	pCD11c^b^	iCD11c^a^	pDC-LAMP^b^	HEV^c^	CD8^a^	PD1^a^	CD4^a^	FoxP3^a^	CD20^a^	CD20 aggr^c^	CD68^a^	CD163^a^
**1**	47.6	70	725.8	30.3	*nd*	*nd*	7	2.3	0.9	1	3.3	0	8.5	3.4
**2**	4.9	100	415.5	13.3	7.7	0	2.4	0	0	0.9	0.2	10	5.9	0.6
**3**	*ne*	5	1108	6.8	*nd*	13	6.1	*nd*	*nd*	0	0	6	9.3	*nd*
**4**	2.5	40	249.3	10.3	10.6	36	0	*ne*	0	0.9	0	6	6.7	1.2
**5**	13.9	15	514.3	8.5	15.1	10	6	0.5	47.5	1	3	10	10	1.3
**6**	1.1	95	554	7	*nd*	52	5.6	3.2	*nd*	0.3	8.2	4	9	0
**7**	20.4	95	360.1	16	*nd*	31	34.5	0	5.4	*Nd*	7.1	3	33.3	0
**8**	0.2	80	692.5	2	29.7	21	4.6	5.6	1.7	2.9	14.9	11	5.9	0
**9**	16.5	100	761.8	31.8	3.6	8	31.3	9.4	0.4	0.7	0.9	8	47.6	5
**10**	14	50	266.5	0	23.5	3	2.1	2.4	0.4	0	0.5	5	3.4	0.7
**11**	17.2	90	502.9	31.8	22.9	21	17.9	4.3	2.4	3.1	7	9	13.2	9.6
**12**	3.2	40	512.5	7.9	*nd*	1	10.5	0	0.9	0.5	5.3	2	22.2	6.2
**13**	1	50	761.6	34	136	27	30.4	0.9	0.5	0.5	4	3	26	0
**14**	29.5	100	263.2	1.6	*nd*	*nd*	1.7	*nd*	0	0	0	0	5.4	*nd*
**15**	10	75	567.9	20.8	21.4	14	19.4	56	0	0.7	3	8	7.5	2
**16**	8.5	50	429.4	11.1	*nd*	*nd*	8.5	2.4	0	0.8	1	3	11.5	1.9
**17**	2.22	95	609.4	16	*35.7*	21	2.2	36	0	5.7	4.6	5	18	8.9
**18**	0.9	60	105.3	0.7	3.3	3	0.8	0	0.3	0.9	0.2	5	0.8	1
**19**	31.6	10	463	3.8	7.2	6	6.3	12.5	0	1.1	1.6	4	23.7	*nd*
**20**	24.3	50	180.6	3.6	*nd*	*nd*	1	*nd*	*nd*	0.8	0	0	8.8	*nd*
**21**	10.4	40	55.4	0	1.4	3	0.9	0	0	3.2	0	3	5.8	*nd*
**22**	27.3	35	249.3	1	*nd*	3	2.3	0	1.3	1.4	0.3	5	25.5	6
**23**	52.5	50	277	8.8	*nd*	0	2.4	3.4	0	4.5	2.6	2	4.9	5.7
**24**	12.9	30	110.8	2	4.3	2	0.6	0	0	3	0	0	12.5	2.7
**25**	8	100	156.3	2	9.8	0	0.3	0	0	2.8	0	1	4.7	1.9
**26**	34.3	70	485.7	0.6	4.5	6	0	0	0	0	0	0	2.9	0.7
**27**	23.3	70	514.2	7	1.5	7	3.1	1.8	0.2	6.1	0.4	2	25	13.5
**28**	20	20	242.6	4.7	3.1	3	3.8	4.6	0.4	1.5	0.3	0	19.8	5.1
**29**	28.4	5	121.3	4.1	9.1	9	2.9	1.1	1.2	1.1	9	9	8.6	5.3

Cell counting was performed in representative fields as described in Methods.

a positive cells/tumor cells*100 (%).

b positive cells/10^5^ µm^2^.

c positive structures/10^5^ µm^2^.

nd, not determined; ne, not evaluable. GO patients are shaded in gray. CD20aggr, CD20 aggregates.

### High tumor proliferation and low tumor HLA-I expression in mTDLN before immunotherapy are associated with a bad outcome for CM patients

Ki-67 measurement was used to determine the proliferation rate of the tumor. The GO cohort had a median Ki-67 positivity of 9.25% (range 0.2–47.6%) and the BO cohort a median positivity of 23.8% (range 0.9–52.5%), the difference being statistically significant (*p=0.037*), implying that Ki-67 positivity is a defining biomarker (**Table 1** and **Figure 2**). HLA-I expression in tumor cells was measured to determine the potential susceptibility of the tumor to be attacked by CD8^+^ cytotoxic lymphocytes, since the lack of surface expression of HLA-I is a common mechanism of resistance (20). The median expression of HLA-I in GO and BO cohorts approached 75% and 40%, respectively, although it did not reach statistical significance (*p=0.056*) (**Table 1** and **Figure 2**). This result implies that even before any treatment was given, BO patients had an incremented number of HLA-I negative cell clones in their tumors. It is striking, however, that two GO patients, #3 and #5, had quite low HLA-I expression (5% and 15% of tumor cells, respectively) (**Table 1** and **Figure 2**). Patient#3 (stage IIIB) had a highly melanotic tumor, was treated with VACCIMEL and survived for 150 months, dying of a heart attack and without evidence of recurrence. How can we, therefore, explain such long DMFS, since this patient had abundant cytotoxic CD8^+^ lymphocytes (numbered as patient #10 in (14)), and the lack of HLA-I would render tumor cells insensitive to CD8^+^ cells attack. Possible explanations could be that since about half of stage III CM patients do not recur even without treatment, i) no micrometastases would be present after mTDLN resection; or ii) a lack of HLA-I would render tumor cells susceptible to NK cells or γδ T cells attack (21). Instead, patient #5 presented a mTDLN with a low HLA-I tumor expression but a high percentage of CD4^+^ T cells (44%) and a high number of CD20^+^ nests in the peritumoral area (**Table 1**) before treatment with interferon alpha-2b. In this patient, no CD8^+^ cell clones were generated after IFNalpha-2b treatment [numbered as patient#20 in (14)]. This patient developed a strong autoimmune response directed to the nervous system, suggesting that the generation of autoantibodies was the cause of such serious adverse effect.

### Examination of immune cell infiltrates shows the presence of HEV-DC-CD62L^+^lymphocytes structures in mTDLN of CM patients with a good outcome following immunotherapeutic treatment

**Figure 3:**
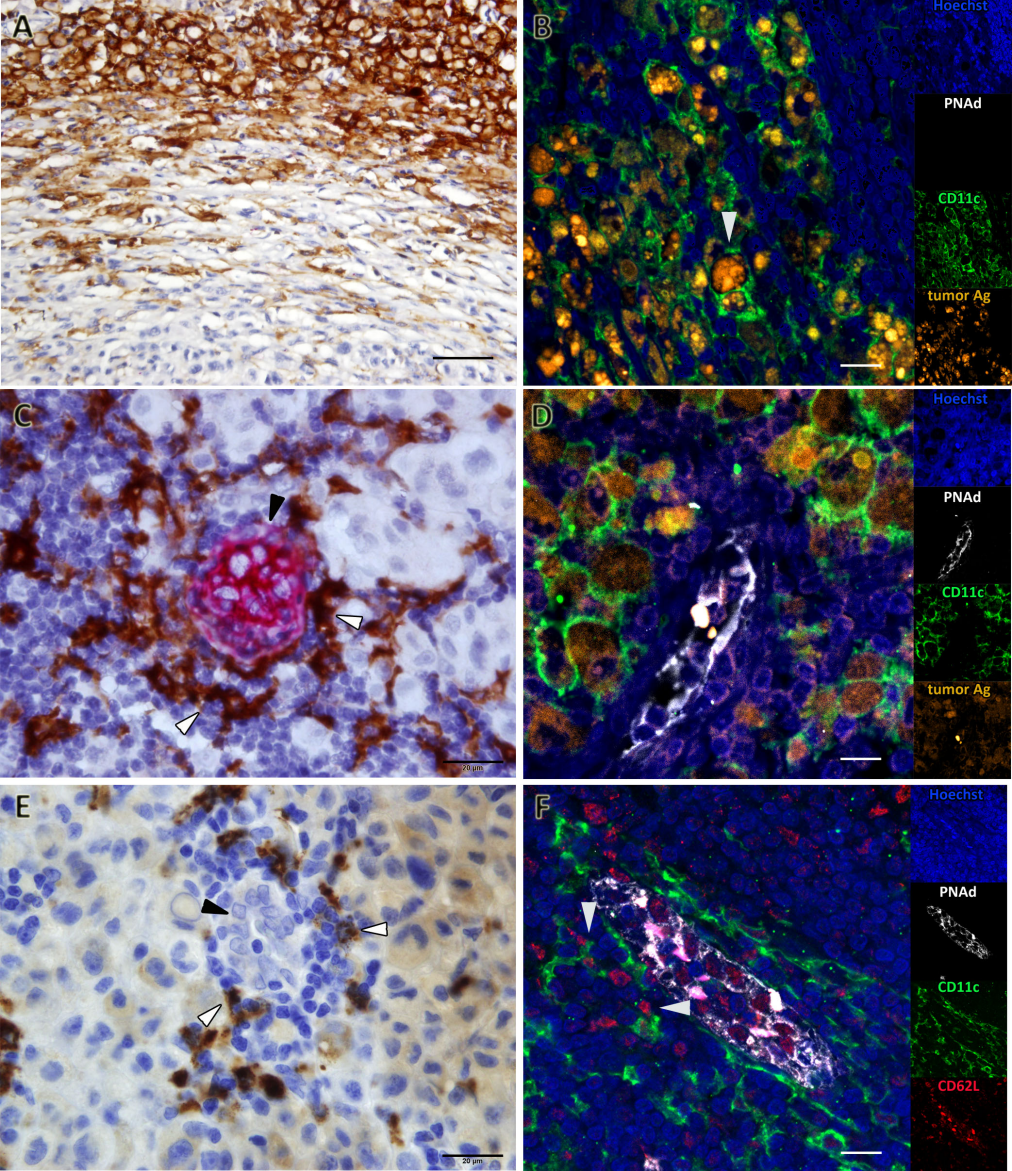
Favoring Antigen-Presenting Structures (FAPS) are present in mTDLN of GO patients. Biomarkers were determined by IHC or multiplex IF, as described under Methods; representative images are shown. **(A)** Patient#17 is enriched in peri- and intra-tumoral CD11c^+^ cells. **(B)** Patient#17 CD11c^+^ cells (green) contain melanin and tyrosinase (orange, white arrow). **(C)** A FAPS example is shown (Patient#13). CD11c^+^ cells (white arrow) are shown surrounding PNAd^+^ HEV (black arrow). **(D)** CD11c^+^ cells (green) surrounding HEV (white) contained melanin and tyrosinase (orange) (Patient#17). **(E)** DC-LAMP^+^ cells (white arrow) surrounding HEV with cuboidal epithelium (black arrow) are shown (Patient#13). **(F)** FAPS structure showing PNAd^+^ (white) HEV interactions between CD62L^+^ cells (red) and CD11c^+^ cells (green) are indicated by arrows (Patient#13). Original magnifications: **(A)** 400X; **(B, D, F)** 200X; **(C, E)** 1000X. Scale bars: **(A)** 50µm; **(B, D, F)** 10µm; **(C, E)** 20µm.

CD11c, DC-LAMP, CD64, CD68 and CD163 biomarkers were measured. For clarity, we describe separately the findings obtained for antigen-presenting cells (APCs) and macrophages of myeloid origin. Concerning APCs, analyses of peri- and intra-tumoral CD11c^+^ demonstrated that these cells are among the most significant immune biomarkers that differentiate GO from BO patients (*p=0.0002* and *p=0.001*, respectively) (**Table 1** and **Figure 2**). CD11c^+^ cells could be found amidst tumor cells or in the peritumoral area. The morphology of CD11c^+^ cells was variable, spanning from large epithelioid-like cells to cells with flattened morphology (**Figure 3A**). We determined that CD11c^+^ cells were able to phagocytose fragments of melanoma cells since abundant tyrosinase and melanin granules were found in their cytoplasm (**Figures 3B, D**); cytoplasmic MART-1 was also detected in these cells (*data not shown*).

HEVs are the entry site of naïve lymphocytes into LN, mainly through the interaction between L-selectin (CD62L) in naïve lymphocytes and Peripheral Node Addressin (PNAd) in cuboidal endothelial cells (22). We investigated whether GO and BO patients differed in HEV numbers. **Table 1** and **Figure 2** show that GO patients had a significantly higher number of HEVs than BO patients (*p=0.004*). Most importantly, we observed in GO patients that some HEVs were surrounded by single to several layers of CD11c^+^ cells and that tyrosinase and melanin granules were detected in the cytoplasm of these APCs (**Figures 3C, D**). Also, peritumoral DC-LAMP^+^ cells, a biomarker for mature DCs, were significantly more abundant in GO that in BO patients (*p=0.001*). Although scarce within tumors, DC-LAMP^+^ cells were detected at the T cell-enriched peritumoral area in large numbers in GO patients (**Supplementary Figures 2A, C**) as compared to BO patients (**Supplementary Figures 2B, D**) and, most significantly, surrounding HEVs (**Figure 3E** and **Supplementary Figure 3**).

**Figure 4:**
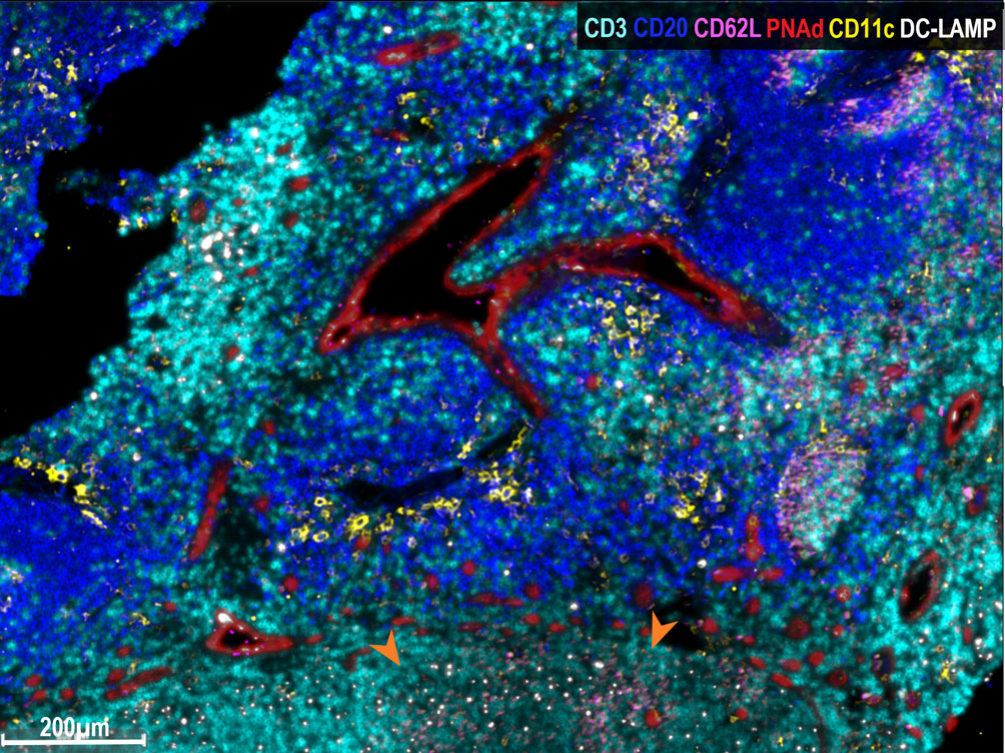
Multiplex immunofluorescence analysis of FAPS. Multiplex staining of a mTDLN peritumoral area of one GO patient (#13) with anti-CD3 (cyan), anti-CD20 (blue), anti-CD62L (pink), anti-PNAd (red), anti-CD11c (yellow), and anti-DC-LAMP (white) antibodies was performed as described under Methods. DC-LAMP^+^ and CD11c^+^ cells surround PNAd^+^ HEV. In the lower part area of the figure, marked with orange arrows, sprouts of PNAd^+^ HEV (red), and CD62L^+^ naïve lymphocytes (pink) making contacts with HEV and DC-LAMP^+^ dendritic cells (white) may be observed.

Finally, we analyzed whether naïve lymphocytes accumulated in the vicinity of HEV-associated CD11c^+^ cells. For this purpose, triple immunostaining was performed with antibodies to PNAd, CD11c and CD62L. Strikingly, one can observe that some CD62L^+^ naïve lymphocytes enter into contact with CD11c^+^ cells, suggesting that immunological synapses are being formed (**Figure 3F**). Thus, we propose that this triad (HEV, Ag-loaded CD11c^+^ cells and CD62L^+^ lymphocytes) constitute a structure that we named *Favoring Antigen-Presenting Structures* (FAPS). **Figure 4** illustrates the proximity of T and B lymphocytes to HEV and CD11c^+^ cells, with DC-LAMP^+^ cells present in the T-cell enriched peritumoral area of one GO patient (#13).

### Macrophage infiltrates in mTDLN exhibit a marked heterogeneity in CM patients before treatment

We also examined the presence of tumor-associated macrophages (TAM) in mTDLN, since their pro- or antitumoral role is highly debated. We first analyzed intratumoral TAM by IHC. CD68^+^ TAM were present with a median of 9.3% in GO patients and 8.7% in BO patients, while CD163^+^ TAM were present with a median of 1% in GO patients and 5% in BO patients; in neither case the differences were statistically significant (**Table 1** and **Figure 2**).

**Figure 5:**
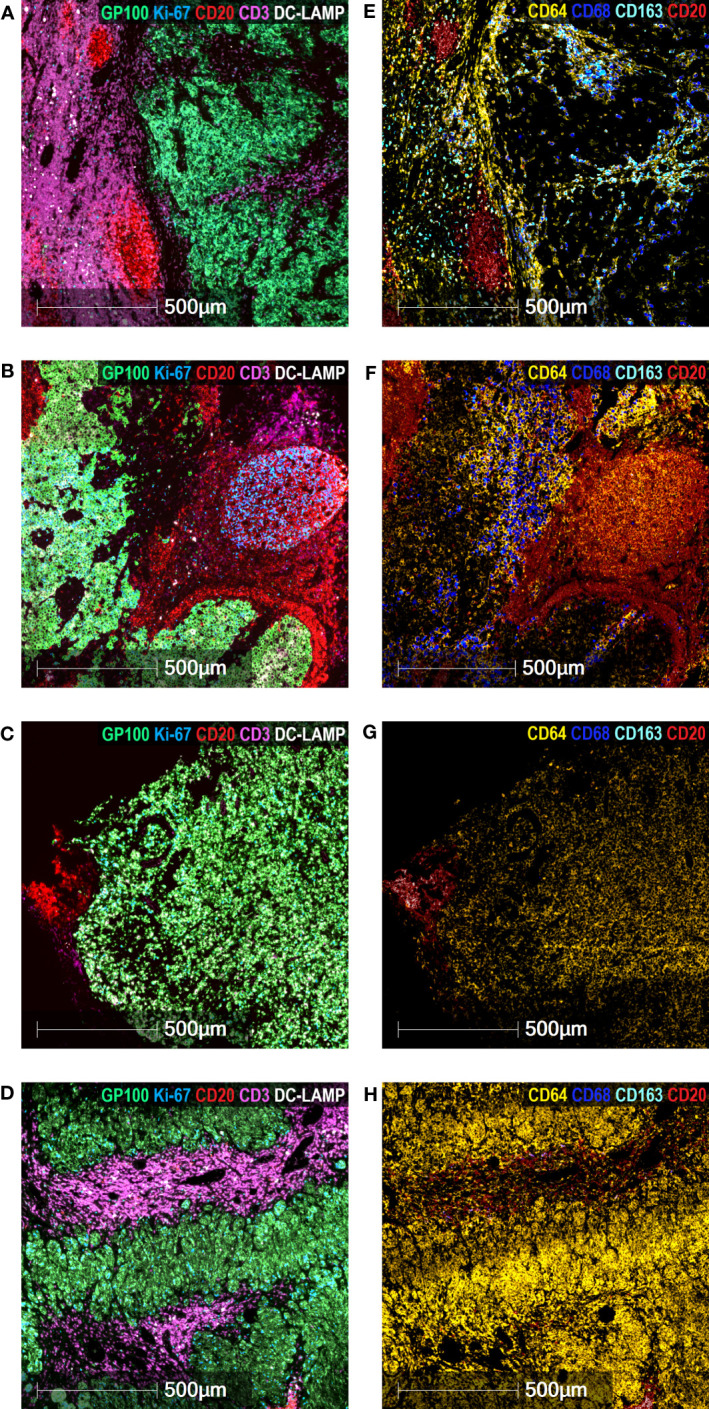
Characterization of TAM in mTDLN. Multiplex Immunofluorescence staining of the mTDLN tumor stroma, tumor nest and peritumoral area of two GO (A&E, patient #2; B&F, patient #17) and two BO (C&G, patient #21; D&H, patient #18) patients was performed as described under Methods. **(A–D)** anti-GP100 (green), anti-Ki-67 (blue), anti-CD20 (red), anti-CD3 (purple), and anti-DC-LAMP (white) antibodies. **(E–H)** anti-CD64 (yellow), anti-CD68 (dark blue), anti-CD163 (cyan) and anti-CD20 (red) antibodies. Scale bars: 500 μm.

We then performed a detailed exploratory analysis of TAM subpopulations in the tumor and peritumoral areas of both GO and BO patients. We included CD64 (FcɣRI) staining, whose expression level on myeloid cells is regulated by IFN-γ. Multiplex immunofluorescence staining was performed in two GO patients (#2, #17) and two BO patients (#18, #21). In GO patient#2, the tumor nest was surrounded by a peritumoral area, in which aggregates of proliferating B cells and a prominent T-cell enriched area were observed (**Figure 5A**). In the tumor nest, both CD64^+^CD68^-^CD163^-^ and CD64^+^CD68^+^CD163^-^ TAM were detected, while CD64^+^CD68^+^CD163^+^ TAM were concentrated within the tumor stroma and the peritumoral area, mostly in the T-cell enriched area (**Figure 5E**). GO Patient#17 exhibited a tumor nest mainly surrounded by a B-cell aggregate (**Figure 5B**). In this case, CD64^+^CD68^+^CD163^-^ TAM were dominant in tumor stroma (**Figure 5F**). Similarly to patient#2, CD64^+^CD68^-^CD163^-^ and CD64^+^CD68^+^CD163^-^ TAM were found in the tumor nest (**Figure 5F**). Strikingly, only few CD64^+^CD68^+^CD163^+^ TAM were located in the peritumoral area (**Figure 5F**).

The images observed in both BO patients were quite different (**Figures 5C, D, G, H**). No intratumoral CD68^+^ nor CD163^+^ TAM was detected (this was coincidently observed by IHC; see **Table 1**). Instead, a high number of intratumoral CD64^+^CD68^-^CD163^-^ TAM was detected in patient#18 (**Figure 5G**); whereas in patient#21, CD64^+^CD68^-^CD163^-^ TAM were found both in tumor and peritumoral areas (**Figure 5H**).

### B-cell aggregates present in mTDLN do not exhibit CD21^+^ follicular dendritic cells

**Figure 6:**
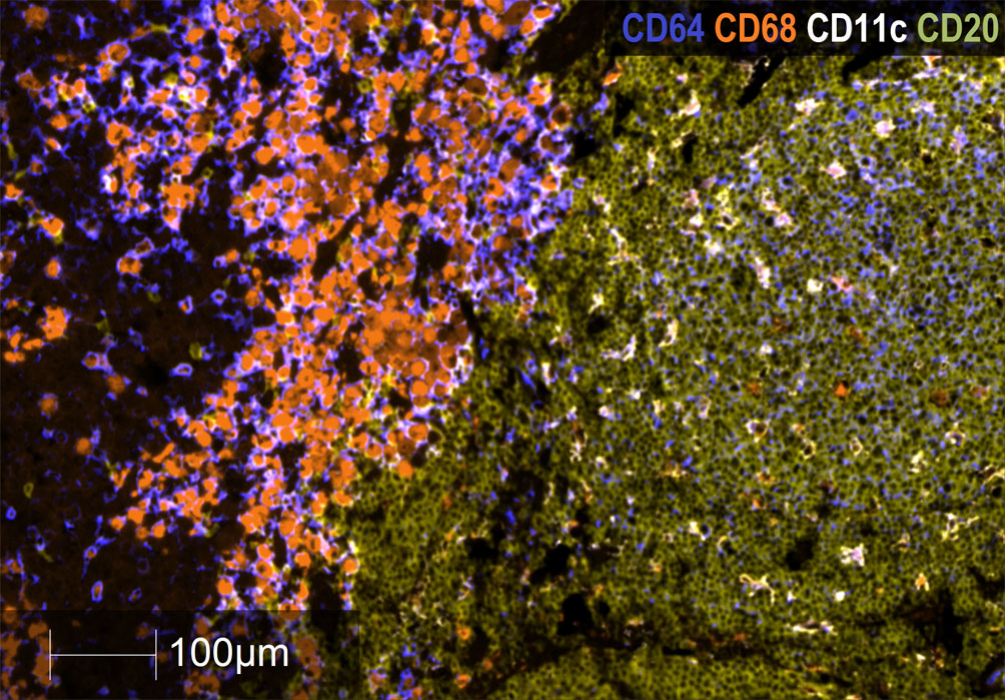
Analysis of B cell CD20^+^ aggregates in mTDLN. Multiplex immunofluorescence staining of a B-cell aggregate of one GO patient (#17) with anti-CD64 (dark blue), anti-CD68 (orange), anti-CD11c (white), and anti-CD20 (khaki) antibodies was performed as described under Methods. Left side of the image: tumor stroma area infiltrated by macrophages; right side of the image: peritumoral area with a large B-cell aggregate infiltrated by CD11c^+^ cells. Most of these cells are also labeled with the anti-CD68 antibody within their cytoplasm and appear as double-labeled (white/orange). Scale Bar: 100μm.

**Figure 7:**
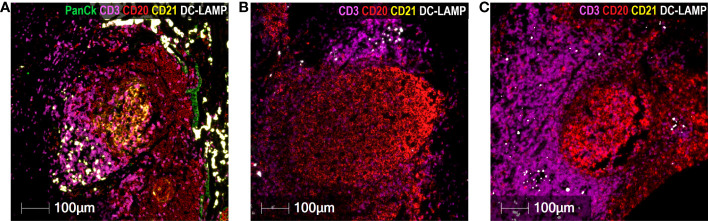
Absence of CD21^+^ FDC in cutaneous melanoma mTDLN. Multiplex immunofluorescence staining was performed with anti-PanCk (green), anti-CD3 (purple), anti-CD20 (red), anti-CD21 (yellow), and anti-DC-LAMP (white) antibodies. **(A)** A lung tumor biopsy was used as positive control and showed a large network of CD21^+^ follicular dendritic cells (FDC) (stained in yellow) within the B-cell area of a tertiary lymphoid structure (TLS). Lack of CD21^+^ Follicular Dendritic Cells in B-cell aggregates of mTDLN are shown in one GO (#17) **(B)** and one BO (#21) **(C)** patients. Scale bars: 100µm.

Regarding CD20^+^ B lymphocytes, they were found either as isolated tumor-infiltrating cells or as small and large aggregates. The number of individual infiltrating CD20^+^ lymphocytes and CD20^+^ aggregates were higher in GO patients than in BO patients (3.0 *vs.* 0.25%; p= 0.027; 5.0 *vs.* 2.0%, p=0.023). A peculiar feature of the mTDLN was the presence of peritumoral aggregates of CD20^+^ B cells in close contact with the tumor itself and with CD64^+^CD68^+^ and CD64^+^CD68^-^ peritumoral TAM (**Figure 6**). In the midst of the B-cell aggregates, CD11c^+^ cells, many of them with CD68^+^ co-expression inside, were observed (**Figure 6**). Strikingly, none of the B-cell aggregates contained detectable CD21^+^ follicular dendritic cells (FDC), in none of the five patients tested (GO patients #2 and #17; BO patients #18, #19 and #21) (**Figures 7B, C**). This is in contrast to lung TLS (tertiary lymphoid structures), where B-cell areas exhibit a network of CD21^+^ FDC, as shown in **Figure 7A**. Lack of CD21^+^ FDC suggests a possible impairment in B-cell follicle function in the mTDLN of cutaneous melanoma patients.

### CD8^+^ lymphocytes present in mTDLN are partially activated.

The presence of brisk tumor-infiltrating CD8^+^ lymphocytes was significantly higher in GO patients than in BO patients (median positivity of 6.1% *vs.* 1.65%, range 0-34.5% *vs*. 0-6.3%), *p=0.004*), as previously reported (23). We also measured PD-1 to inquire whether some CD8^+^ cells had been activated. Although no differences were found between GO and BO patients, when present, PD-1^+^ lymphocytes were located mainly in the peritumoral area adjacent to the tumor nests and infiltrating only the peripheral tumor cell layers (**Supplementary Figure 4**).

Regarding CD4^+^ lymphocytes, no difference between GO and BO patients was detected. However, FoxP3^+^ lymphocytes were more abundant in BO patients than in GO patients (median positivity of 1.45% *vs.* 0.75%, respectively; *p=0.021*) (**Figure 2**).

### Vaccination in GO patients induce higher immune response than in BO patients

To investigate if the more favorable cellular and structural characteristics in mTDLN in GO patients correlated with a higher immune response, we examined the response to VACCIMEL Ags by ELISPOT in vaccinated patients of both cohorts. The examined patients and data were extracted from the CASVAC 0401 study (13). In vaccinated patients (**Supplementary Figure 5A**), 7/17 patients from the GO cohort and 5/12 patients from the BO cohort were analyzed, showing that pre-treatment reactivity was detected in only 2/7 GO patients and in 1/5 BO patients. After vaccination, reactive clones increased in 6/7 (86%) patients from the GO cohort and in 2/5 (40%) patients from the BO cohort. In the two IFN-treated patients (**Supplementary Figure 5B**), neither basal reactivity nor reactivity 6 months after treatment were detected. Although the differences do not attain statistical significance due to the small number of cases, a tendency in favor of a higher immune response in the GO cohort appears possible.

## Discussion

The human genome contains about 7.5% of its genes coding for proteins related to the immune system (24). It is therefore not surprising that the immune system functionality may vary between individuals in many diseases, and this also applies to the immune response to cancer. This issue has become particularly important since manipulation of the IS has led to major advances in cancer treatment, and cutaneous melanoma (CM) has been a paradigmatic disease for the development of immunotherapies (25). The variety of clinical responses to diverse IT in CM has long been known. For example, high-dose IL-2 produced meaningful clinical responses in only 17% of the CM-treated patients (26). Adjuvant therapy with high-dose IFN-α2b prolonged disease-free intervals in only a small subset of CM patients (27). Also, a clinical trial in adjuvancy with anti-PD-1 pembrolizumab or placebo (EORTC 1325-MG/KEYNOTE-054) in stage III CM patients demonstrated, after a median follow-up of 42.3 months, that only 15% of the patients derived benefit from the treatment, since about 50% of the placebo arm patients did not progress, and around 35% of the patients did not increase DMFS (6).

Therefore, we need a better knowledge of both actors of the disease, the tumor and the IS, to select more appropriate treatments. For this purpose, we centered our studies on patients who had entered clinical trials dedicated to studying the effect of VACCIMEL, an allogeneic vaccine adjuvanted with BCG and GM-CSF (14). From this population, we selected two sets of patients: those with long DMFS (GO patients) and those with very short DMFS (BO patients). We analyzed mTDLN since they are the first site of encounter between metastatic cells from the primary tumor and an organized lymphoid structure. Besides, lymphadenectomy was mandatory prior to inclusion in the vaccine trial. To analyze the FFPE biopsies, we performed IHC and multiplex IF. When both cohorts were compared, significant differences were found. An important finding was that GO patients were heavily infiltrated by CD11c^+^ cells, whereas BO patients were practically devoid of them. GO patients also had more abundant PNAd^+^ HEVs and, most importantly, CD11c^+^ cells and DC-LAMP^+^ cells surrounding HEVs.

We describe to our knowledge for the first time a triad structure that could contribute to a stronger immune reactivity against tumors: the “Favoring Antigen-Presenting Structures (FAPS)”. This triad is formed by Ag-loaded DC, HEV and naïve lymphocytes establishing synaptic contacts with DC. Of note, HEV and FAPS were hardly found in BO patients. As to the analysis of the three components of the triad, DCs are the most efficient APC whose three main capabilities are to capture, process and present exogenous and endogenous antigens to cognate TCR CD4^+^ or CD8^+^ lymphocytes (28). We detected DC mainly by CD11c integrin and DC-LAMP expression. CD11c plays a fundamental role in DC physiology. Wang et al. found that blocking CD11c diminishes acute graft-versus-host disease in mice allogeneic bone marrow transplantation (29). Besides, CD11c would also intervene in the capture of CD47-deficient cells, CD47 being a “don´t eat me” molecule (30). It is important to decipher CD11c functional state, since Probst et al. demonstrated that resting CD11c^+^ cells induce tolerance whereas activated CD11c^+^ cells are capable of inducing effector CD8^+^ and CD4^+^ T lymphocytes (31). DC-LAMP^+^ cells are generally assumed to represent mature DCs (32). The question that should be asked is whether the CD11c^+^ cells found in mTDLN of GO patients are immature DCs, waiting for maturation stimuli to act as fully developed APCs, or whether they are already able to present Ag. To support the latter possibility, Pizzurro et al. demonstrated that CD11c^+^ cells, which phagocytosed tumor Ag, were able to stimulate IFN-gamma production of a specific T-cell clone (19). Besides, Barrio et al. demonstrated that DC or activated TAM are equally capable of cross-presenting MART-1 Ag to CD8^+^ T cells (33). It has also been reported by van de Hout et al. a sequential suppression of different DC subsets in CM sentinel LN (34). Another component of the triad, HEVs, are highly specialized post-capillary venules, endowed with a cuboid epithelium that expresses an ensemble of glycoproteins such as Peripheral Node Addressin (PNAd), a putative ligand of L-selectin (CD62L). Moussion and Girard demonstrated in mice that CD11c^+^ cells are essential for the formation of HEVs (35). Therefore, we suggest that, in GO patients, a higher number of CD11c^+^ cells could trigger the formation of more HEVs than in BO patients. Concerning the possible role of HEVs in cancer, Martinet et al. showed in primary CM that the number of HEVs was related to a good prognosis (36). On the contrary, in BO patients HEV would have been impacted by tumor-induced LN remodeling. The third component of the triad, the naïve lymphocytes, express L-selectin (CD62L), which binds to various sulfated syalyl LeX - containing molecules present in HEVs (37). Thus, we propose that FAPS are structures in which emerging naïve lymphocytes would make synaptic contact with Ag-loaded DC, thus being primed as soon as they leave HEVs; those naïve lymphocytes that fail priming at this instance would be later primed inside the tumor after encountering intratumoral DCs.

Emerging data support a model where LN architecture is progressively disrupted by tumors, leading to profound changes in immune cell function that may contribute to metastatic potential (38). Jones et al. revealed LN remodeling by tumor growth characterized by HEV dilation and dedifferentiation, with loss of PNAd expression and function (39). Our results partially contradict the findings of Yaddanapudi et al., who concluded that an immunosuppressive TME is predominant in sentinel LN (10). Although immunosuppression may be more frequent and permanent in BO patients, we suggest that, in selected GO patients, the Ag-presentation machinery was present and ready to be triggered by subsequent vaccination. The number and quality of DCs in mTDLN could also modulate the number of antitumor reactive T cell clones and the subsequent effect of immunotherapies. However, what could be the relationship between what is observed in an already excised mTDLN and the future evolution of the patients to adjuvant immunotherapy? Vaccination after mTDLN |resection may boost immune responses in other uncompromised LN. In a sequence of studies on VACCIMEL, we found that, in most patients, there was no pre-treatment basal reactivity against melanoma Ags or neoantigens, but such reactivity was triggered after vaccination, demonstrating a competent IS (40, 41).

Another finding of this work was that GO patients had a significantly higher CD8^+^ lymphocytic infiltration, confirming previous results (23). PD-1 expression was restricted to a small number of CD8^+^ lymphocytes, suggesting that only a few CD8^+^ cells had been primed by their cognate antigens (42), thus making infiltration and priming two different processes. Combining the findings of CD8^+^ lymphocytes and the abundance of CD11c^+^ cells in GO patients, it could also be hypothesized that CD8^+^ cells lack the “effector stimulus” provided by fully mature DC. Along this line, Makedonas et al. demonstrated in a model of influenza infection in mice, that the effector phase of CD8^+^ lymphocytes needs Ag presentation by DC and CD28 co-stimulation for sustaining activity (43). Another possibility is that the tumor immunosuppression established in the mTDLN, as previously reported (44), inhibits primed CD8^+^ lymphocytes to become fully active and later exhausted, even in GO patients. Indeed, the dynamics of CD8^+^ T cell responses to cancer immunotherapy was found to be disrupted in human mTDLN (45). Also, we found more Foxp3^+^ T regulatory cells (Treg) in BO patients. Beyond the negative regulatory function of Treg on effector T lymphocytes, it has recently been reported that depletion of Treg can induce HEV neogenesis through an alternative mechanism involving CD8^+^ cells activation, different from HEV ontogenic development (46). Thus, in a context of strong immune stimulation with a vaccine and a Th1 polarizing adjuvant (i.e. VACCIMEL plus BCG), some CM patients who are capable of developing FAPS in their LN could generate a potent immune response associated with GO. Other patients may have an impaired capacity to develop such structures and thus could be less efficient in mounting effective priming upon vaccination.

Regarding CD20^+^ B lymphocytes, they were mostly located in proliferating aggregates (as shown by the double positive staining with CD20 and Ki-67) and close to the tumor or marginally within it (**Figures 1**, **5**–**7**). CD20^+^ aggregates were preferentially found in GO patients. Strikingly, a lack of CD21^+^ FDC network as shown by the absence of CD21 and the presence of CD11c^+^ cells in GO and BO patients suggest a disruption in B-cell function in mTDLN, although the presence of CD21^-^ FDC cannot be excluded. Of note, the role of CD20^+^ aggregates remains unknown, although most of them are clearly different from the mature TLS described in several tumors such as lung carcinomas (47).

The role of TAM in cancer has been extensively studied and debated, and two extreme types of TAM have been described: M1, which is detrimental to tumors; and M2, which is immunosuppressive and, therefore, induces tolerance and is often associated with a bad prognosis (48). However, transcriptomic analyses have revealed that TAM have a mixed phenotype (49). In a first approach, we used CD68, a type I glycosylated lysosomal membrane protein, to determine the whole TAM population (50). CD163, a glucocorticoid-inducible member of the scavenger receptor cysteine-rich family, expressed in innate immune cell populations (51), was used to detect M2 TAM (52). Neither the percentages of CD68^+^ nor CD163^+^ TAM attained statistical significance between GO and BO patients. An exploratory multiplex study performed on four patients revealed the extreme plasticity of TAM. Thus, CD68^+^CD64^+^ cells were only found in GO patients. Since myeloid cells express CD64 (FcyRI), a high-affinity receptor, the phagocytic role of tumor cells opsonized by locally produced Abs should be studied. Of note, CD64^+^ TAM were highly expressed in BO patients, both in intra-and peri-tumoral areas. Recently, Martinek et al. analyzed CM metastasis and found that CD14^+^ TAM displayed different signatures in tumor nests from those in tumor stroma associated with long-term survival (53).

It should be emphasized that both cohorts received adjuvant IT, since 22/29 patients were treated with VACCIMEL and 6/29 patients received IFN-α2b.

We have previously shown that peripheral lymphocytes, which in most patients were non-reactive to tumor-associated antigens and/or neoantigens prior to vaccination, became reactive after treatment with BCG-containing VACCIMEL, thus pointing to an increased number or awakening of reactive T-cell clones (14, 41). We provide here preliminary evidence that most analyzed GO patients (6/7 tested) reacted to vaccination by producing a larger number of CD8 lymphocyte clones against melanoma antigens than BO patients. However, some BO patients, (2/5 tested) who also produced reactive clones against melanoma antigens, failed to control the disease, probably due to a highly proliferating tumor, one of the ominous characteristics of melanoma.

Although radical lymphadenectomy is no longer mandatory in stage III CM patients, sentinel TDLN exeresis is still performed in patients with thick primary melanomas (54) and radical LN resection may be performed in more advanced stage III patients. It will be of interest to study prospectively whether the analysis here described on mTDLN may predict the evolution and response to different treatments.

## Data availability statement

The original contributions presented in the study are included in the article/**Supplementary Material**. Further inquiries can be directed to the corresponding author.

## Ethics statement

The studies involving humans were approved by Ethics Committee of the Alexander Fleming Institute (Buenos Aires, Argentina). The studies were conducted in accordance with the local legislation and institutional requirements. The participants provided their written informed consent to participate in this study.

## Author contributions

JM: conception and design, collection and assembly of data, and manuscript writing. MA, MB and MP: collection and assembly of data, data analysis and interpretation, and manuscript writing. J-LT and M-CD-N: data interpretation and manuscript writing. AB and MPo: collection and assembly of data, data analysis and interpretation. AB: medical pathologist (Argentina); MA: Associate Researcher from CONICET (Argentina); MPa: PhD student (France); MPo: graduate student (Argentina); M-CD-N: Research Director at Inserm and Head of laboratory (France); J-LT: Research Director emeritus at Inserm (France); MB: Independent Researcher from CONICET and sub-Head of laboratory (Argentina); JM: Superior Researcher emeritus from CONICET and Head of laboratory (Argentina). All authors contributed to the article and approved the submitted version.

## References

[1] Dummer R Hauschild A Santinami M Atkinson V Mandalà M Kirkwood JM. Five-year analysis of adjuvant dabrafenib plus trametinib in stage III melanoma. N Engl J Med (2020) 383:1139–48. doi: 10.1056/NEJMoa2005493 32877599

[2] Villanueva J Vultur A Herlyn M. Resistance to BRAF inhibitors: unraveling mechanisms and future treatment options. Cancer Res (2011) 71:7137–40. doi: 10.1158/0008-5472.CAN-11-1243 PMC3588168 22131348

[3] Leach DR Krummel MF Allison JP. Enhancement of antitumor immunity by CTLA-4 blockade. Science (1996) 271:1734–6. doi: 10.1126/science.271.5256.1734 8596936

[4] Iwai Y Ishida M Tanaka Y Okazaki T Honjo T Minato N. Involvement of PD-L1 on tumor cells in the escape from host immune system and tumor immunotherapy by PD-L1 blockade. Proc Natl Acad Sci USA (2002) 99:12293–7. doi: 10.1073/pnas.192461099 PMC129438 12218188

[5] Ascierto PA Del Vecchio M Mandalá M Gogas H Arance AM Dalle S. Adjuvant nivolumab versus ipilimumab in resected stage IIIB-C and stage IV melanoma (CheckMate 238): 4-year results from a multicentre, double-blind, randomised, controlled, phase 3 trial. Lancet Oncol (2020) 21:1465–77. doi: 10.1016/S1470-2045(20)30494-0 32961119

[6] Eggermont AMM Blank CU Mandalà M Long GV Atkinson VG Dalle S. Adjuvant pembrolizumab versus placebo in resected stage III melanoma (EORTC 1325-MG/KEYNOTE-054): distant metastasis-free survival results from a double-blind, randomised, controlled, phase 3 trial. Lancet Oncol (2021) 22:643–54. doi: 10.1016/S1470-2045(21)00065-6 33857412

[7] Sivendran S Chang R Pham L Phelps RG Harcharik ST Hall LD. Dissection of immune gene networks in primary melanoma tumors critical for antitumor surveillance of patients with stage II-III resectable disease. J Invest Dermatol (2014) 134:2202–11. doi: 10.1038/jid.2014.85 PMC4291112 24522433

[8] Erdag G Schaefer JT Smolkin ME Deacon DH Shea SM Dengel LT. Immunotype and immunohistologic characteristics of tumor-infiltrating immune cells are associated with clinical outcome in metastatic melanoma. Cancer Res (2012) 72:1070–80. doi: 10.1158/0008-5472.CAN-11-3218 PMC3306813 22266112

[9] Bogunovic D O’Neill DW Belitskaya-Levy I Vacic V Yu Y-L Adams S. Immune profile and mitotic index of metastatic melanoma lesions enhance clinical staging in predicting patient survival. Proc Natl Acad Sci (2009) 106:20429–34. doi: 10.1073/pnas.0905139106 PMC2787158 19915147

[10] Yaddanapudi K Stamp BF Subrahmanyam PB Smolenkov A Waigel SJ Gosain R. Single-cell immune mapping of melanoma sentinel lymph nodes reveals an actionable immunotolerant microenvironment. Clin Cancer Res (2022) 28:2069–81. doi: 10.1158/1078-0432.CCR-21-0664 PMC9840851 35046061

[11] Barrio MM de Motta PT Kaplan J von Euw EM Bravo AI Chacón RD. A phase I study of an allogeneic cell vaccine (VACCIMEL) with GM-CSF in melanoma patients. J Immunother (2006) 29:444–54. doi: 10.1097/01.cji.0000208258.79005.5f 16799340

[12] von Euw EM Barrio MM Furman D Levy EM Bianchini M Peguillet I. A phase I clinical study of vaccination of melanoma patients with dendritic cells loaded with allogeneic apoptotic/necrotic melanoma cells. Analysis of toxicity and immune response to the vaccine and of IL-10 -1082 promoter genotype as predictor of disease progression. J Transl Med (2008) 6:6. doi: 10.1186/1479-5876-6-6 18221542 PMC2265680

[13] Mordoh J Kairiyama C Bover L Solarolo E. Allogeneic cells vaccine increases disease-free survival in stage III melanoma patients. A non randomized phase II study. Medicina (B Aires) (1997) 57:421–7. 9674264

[14] Mordoh J Pampena MB Aris M Blanco PA Lombardo M von Euw EM. Phase II study of adjuvant immunotherapy with the CSF-470 vaccine plus bacillus calmette-guerin plus recombinant human granulocyte macrophage-colony stimulating factor vs medium-dose interferon alpha 2B in stages IIB, IIC, and III cutaneous melanoma patients: A single institution, randomized study. Front Immunol (2017) 8:625. doi: 10.3389/fimmu.2017.00625 28620382 PMC5449770

[15] Mordoh A Aris M Carri I Bravo AI Podaza E Pardo JCT. An update of cutaneous melanoma patients treated in adjuvancy with the allogeneic melanoma vaccine VACCIMEL and presentation of a selected case report with in-transit metastases. Front Immunol (2022) 13:842555. doi: 10.3389/fimmu.2022.842555 35432383 PMC9011367

[16] Aris M Bravo AI Barrio MM Mordoh J. Inoculation site from a cutaneous melanoma patient treated with an allogeneic therapeutic vaccine: a case report. Front Immunol (2015) 6:144. doi: 10.3389/fimmu.2015.00144 25870600 PMC4378302

[17] Tarhini AA Lee SJ Tan A-C El Naqa IM Stephen Hodi F Butterfield LH. Improved prognosis and evidence of enhanced immunogenicity in tumor and circulation of high-risk melanoma patients with unknown primary. J Immunother Cancer (2022) 10:e004310. doi: 10.1136/jitc-2021-004310 35074904 PMC8788316

[18] Devi-Marulkar P Fastenackels S Karapentiantz P Goc J Germain C Kaplon H. Regulatory T cells infiltrate the tumor-induced tertiary lymphoïd structures and are associated with poor clinical outcome in NSCLC. Commun Biol (2022) 5:1416. doi: 10.1038/s42003-022-04356-y 36566320 PMC9789959

[19] Pizzurro GA Tapia IJ Sganga L Podhajcer OL Mordoh J Barrio MM. Cytokine-enhanced maturation and migration to the lymph nodes of a human dying melanoma cell-loaded dendritic cell vaccine. Cancer Immunol Immunother (2015) 64:1393–406. doi: 10.1007/s00262-015-1743-z PMC11028647 26197849

[20] Ladányi A Hegyi B Balatoni T Liszkay G Rohregger R Waldnig C. HLA class I downregulation in progressing metastases of melanoma patients treated with ipilimumab. Pathol Oncol Res (2022) 28:1610297. doi: 10.3389/pore.2022.1610297 35531074 PMC9073691

[21] de Vries NL van de Haar J Veninga V Chalabi M Ijsselsteijn ME van der Ploeg M. γδ T cells are effectors of immunotherapy in cancers with HLA class I defects. Nature (2023) 613:743–50. doi: 10.1038/s41586-022-05593-1 PMC9876799 36631610

[22] Blanchard L Girard J-P. High endothelial venules (HEVs) in immunity, inflammation and cancer. Angiogenesis (2021) 24:719–53. doi: 10.1007/s10456-021-09792-8 PMC8487881 33956259

[23] Rao UNM Lee SJ Luo W Mihm MC Kirkwood JM. Presence of tumor-infiltrating lymphocytes and a dominant nodule within primary melanoma are prognostic factors for relapse-free survival of patients with thick (t4) primary melanoma: pathologic analysis of the e1690 and e1694 intergroup trials. Am J Clin Pathol (2010) 133:646–53. doi: 10.1309/AJCPTXMEFOVYWDA6 PMC3586796 20231618

[24] Kelley J de Bono B Trowsdale J. IRIS: a database surveying known human immune system genes. Genomics (2005) 85:503–11. doi: 10.1016/j.ygeno.2005.01.009 15780753

[25] Huang AC Zappasodi R. A decade of checkpoint blockade immunotherapy in melanoma: understanding the molecular basis for immune sensitivity and resistance. Nat Immunol (2022) 23:660–70. doi: 10.1038/s41590-022-01141-1 PMC9106900 35241833

[26] Rosenberg SA Yang JC Topalian SL Schwartzentruber DJ Weber JS Parkinson DR. Treatment of 283 consecutive patients with metastatic melanoma or renal cell cancer using high-dose bolus interleukin 2. JAMA (1994) 271:907–13. doi: 10.1001/jama.1994.03510360033032 8120958

[27] Kirkwood JM Strawderman MH Ernstoff MS Smith TJ Borden EC Blum RH. Interferon alfa-2b adjuvant therapy of high-risk resected cutaneous melanoma: the eastern cooperative oncology group trial EST 1684. J Clin Oncol (2023) 41:425–35. doi: 10.1200/JCO.22.02264 36649675

[28] Hubert M Gobbini E Bendriss-Vermare N Caux C Valladeau-Guilemond J. Human tumor-infiltrating dendritic cells: from in situ visualization to high-dimensional analyses. Cancers (Basel) (2019) 11:1082. doi: 10.3390/cancers11081082 31366174 PMC6721288

[29] Wang Q Su X He Y Wang M Yang D Zhang R. CD11c participates in triggering acute graft-versus-host disease during bone marrow transplantation. Immunology (2021) 164:148–60. doi: 10.1111/imm.13350 PMC8358721 33934334

[30] Wu J Wu H An J Ballantyne CM Cyster JG. Critical role of integrin CD11c in splenic dendritic cell capture of missing-self CD47 cells to induce adaptive immunity. Proc Natl Acad Sci USA (2018) 115:6786–91. doi: 10.1073/pnas.1805542115 PMC6042080 29891680

[31] Probst HC van den Broek M. Priming of CTLs by lymphocytic choriomeningitis virus depends on dendritic cells. J Immunol (2005) 174:3920–4. doi: 10.4049/jimmunol.174.7.3920 15778347

[32] de Saint-Vis B Vincent J Vandenabeele S Vanbervliet B Pin JJ Aït-Yahia S. A novel lysosome-associated membrane glycoprotein, DC-LAMP, induced upon DC maturation, is transiently expressed in MHC class II compartment. Immunity (1998) 9:325–36. doi: 10.1016/s1074-7613(00)80615-9 9768752

[33] Barrio MM Abes R Colombo M Pizzurro G Boix C Roberti MP. Human macrophages and dendritic cells can equally present MART-1 antigen to CD8(+) T cells after phagocytosis of gamma-irradiated melanoma cells. PloS One (2012) 7:e40311. doi: 10.1371/journal.pone.0040311 22768350 PMC3388056

[34] van den Hout MFCM Koster BD Sluijter BJR Molenkamp BG van de Ven R van den Eertwegh AJM. Melanoma sequentially suppresses different DC subsets in the sentinel lymph node, affecting disease spread and recurrence. Cancer Immunol Res (2017) 5:969–77. doi: 10.1158/2326-6066.CIR-17-0110 28935649

[35] Moussion C Girard J-P. Dendritic cells control lymphocyte entry to lymph nodes through high endothelial venules. Nature (2011) 479:542–6. doi: 10.1038/nature10540 22080953

[36] Martinet L Le Guellec S Filleron T Lamant L Meyer N Rochaix P. High endothelial venules (HEVs) in human melanoma lesions: Major gateways for tumor-infiltrating lymphocytes. Oncoimmunology (2012) 1:829–39. doi: 10.4161/onci.20492 PMC3489738 23162750

[37] Leppänen A Parviainen V Ahola-Iivarinen E Kalkkinen N Cummings RD. Human L-selectin preferentially binds synthetic glycosulfopeptides modeled after endoglycan and containing tyrosine sulfate residues and sialyl Lewis x in core 2 O-glycans. Glycobiology (2010) 20:1170–85. doi: 10.1093/glycob/cwq083 PMC2948818 20507883

[38] du Bois H Heim TA Lund AW. Tumor-draining lymph nodes: At the crossroads of metastasis and immunity. Sci Immunol (2021) 6:eabg3551. doi: 10.1126/sciimmunol.abg3551 34516744 PMC8628268

[39] Jones D Wang Z Chen IX Zhang S Banerji R Lei P-J. Solid stress impairs lymphocyte infiltration into lymph-node metastases. Nat BioMed Eng (2021) 5:1426–36. doi: 10.1038/s41551-021-00766-1 PMC8678215 34282290

[40] Aris M Bravo AI Garcia Alvarez HM Carri I Podaza E Blanco PA. Immunization With the CSF-470 Vaccine Plus BCG and rhGM-CSF Induced in a Cutaneous Melanoma Patient a TCRβ Repertoire Found at Vaccination Site and Tumor Infiltrating Lymphocytes That Persisted in Blood. Front Immunol (2019) 10:2213. doi: 10.3389/fimmu.2019.02213 31620131 PMC6759869

[41] Podaza E Carri I Aris M von Euw E Bravo AI Blanco P. Evaluation of T-cell responses against shared melanoma associated antigens and predicted neoantigens in cutaneous melanoma patients treated with the CSF-470 allogeneic cell vaccine plus BCG and GM-CSF. Front Immunol (2020) 11:1147. doi: 10.3389/fimmu.2020.01147 32582212 PMC7290006

[42] Lee J Ahn E Kissick HT Ahmed R. Reinvigorating exhausted T cells by blockade of the PD-1 pathway. For Immunopathol Dis Therap (2015) 6:7–17. doi: 10.1615/ForumImmunDisTher.2015014188 PMC5341794 28286692

[43] Makedonas G Hutnick N Haney D Amick AC Gardner J Cosma G. Perforin and IL-2 upregulation define qualitative differences among highly functional virus-specific human CD8 T cells. PloS Pathog (2010) 6:e1000798. doi: 10.1371/journal.ppat.1000798 20221423 PMC2832688

[44] van Krimpen A Gerretsen VIV Mulder EEAP van Gulijk M van den Bosch TPP von der Thüsen J. Immune suppression in the tumor-draining lymph node corresponds with distant disease recurrence in patients with melanoma. Cancer Cell (2022) 40:798–9. doi: 10.1016/j.ccell.2022.06.009 35839777

[45] Rahim MK Okholm TLH Jones KB McCarthy EE Liu CC Yee JL. Dynamic CD8+ T cell responses to cancer immunotherapy in human regional lymph nodes are disrupted in metastatic lymph nodes. Cell (2023) 186:1127–1143.e18. doi: 10.1016/j.cell.2023.02.021 36931243 PMC10348701

[46] Colbeck EJ Jones E Hindley JP Smart K Schulz R Browne M. Treg depletion licenses T cell-driven HEV neogenesis and promotes tumor destruction. Cancer Immunol Res (2017) 5:1005–15. doi: 10.1158/2326-6066.CIR-17-0131 PMC5668144 28947544

[47] Domblides C Rochefort J Riffard C Panouillot M Lescaille G Teillaud J-L. Tumor-associated tertiary lymphoid structures: from basic and clinical knowledge to therapeutic manipulation. Front Immunol (2021) 12:698604. doi: 10.3389/fimmu.2021.698604 34276690 PMC8279885

[48] Ruffell B Coussens LM. Macrophages and therapeutic resistance in cancer. Cancer Cell (2015) 27:462–72. doi: 10.1016/j.ccell.2015.02.015 PMC4400235 25858805

[49] Lawrence T Natoli G. Transcriptional regulation of macrophage polarization: enabling diversity with identity. Nat Rev Immunol (2011) 11:750–61. doi: 10.1038/nri3088 22025054

[50] Chistiakov DA Killingsworth MC Myasoedova VA Orekhov AN Bobryshev YV. CD68/macrosialin: not just a histochemical marker. Lab Invest (2017) 97:4–13. doi: 10.1038/labinvest.2016.116 27869795

[51] Schaer DJ Schaer CA Buehler PW Boykins RA Schoedon G Alayash AI. CD163 is the macrophage scavenger receptor for native and chemically modified hemoglobins in the absence of haptoglobin. Blood (2006) 107:373–80. doi: 10.1182/blood-2005-03-1014 16189277

[52] Wang H Hu W-M Xia Z-J Liang Y Lu Y Lin S-X. High numbers of CD163+ tumor-associated macrophages correlate with poor prognosis in multiple myeloma patients receiving bortezomib-based regimens. J Cancer (2019) 10:3239–45. doi: 10.7150/jca.30102 PMC6603386 31289595

[53] Martinek J Lin J Kim KI Wang VG Wu T-C Chiorazzi M. Transcriptional profiling of macrophages in situ in metastatic melanoma reveals localization-dependent phenotypes and function. Cell Rep Med (2022) 3:100621. doi: 10.1016/j.xcrm.2022.100621 35584631 PMC9133468

[54] KudChadkar RR Michielin O van Akkooi ACJ. Practice-changing developments in stage III melanoma: surgery, adjuvant targeted therapy, and immunotherapy. Am Soc Clin Oncol Educ Book (2018) 38:759–62. doi: 10.1200/EDBK_200241 30231370

