# Supplementary material for: HEV-associated dendritic cells are observed in metastatic tumor-draining lymph nodes of cutaneous melanoma patients with longer distant metastasis-free survival after adjuvant immunotherapy
Source: Front Immunol. 2023 Aug 25;14:1231734. doi: 10.3389/fimmu.2023.1231734 (PMC10485604; doi:10.3389/fimmu.2023.1231734)
Supplement: Supplementary file 1 [file Image_1.pdf]

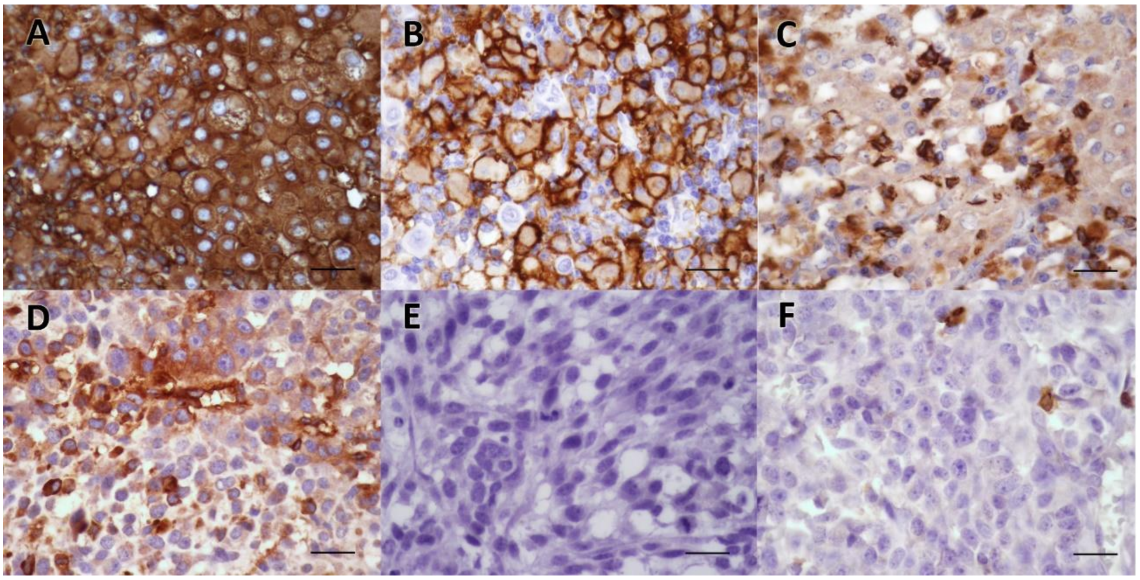

Supplementary Figure 1. Representative immunostaining of intratumoral biomarkers in CM mTDLN. GO patients (A, B, C) and BO patients (D, E, F) are shown. **A and D**: HLA-I staining; **B and E**: CD11c staining; **C and F**: CD8 staining. IHC was performed as described under Methods. Original magnification: 400X. Scale bars: 50 µm.
