# Supplementary material for: HEV-associated dendritic cells are observed in metastatic tumor-draining lymph nodes of cutaneous melanoma patients with longer distant metastasis-free survival after adjuvant immunotherapy
Source: Front Immunol. 2023 Aug 25;14:1231734. doi: 10.3389/fimmu.2023.1231734 (PMC10485604; doi:10.3389/fimmu.2023.1231734)
Supplement: Supplementary file 2 [file Image_2.pdf]

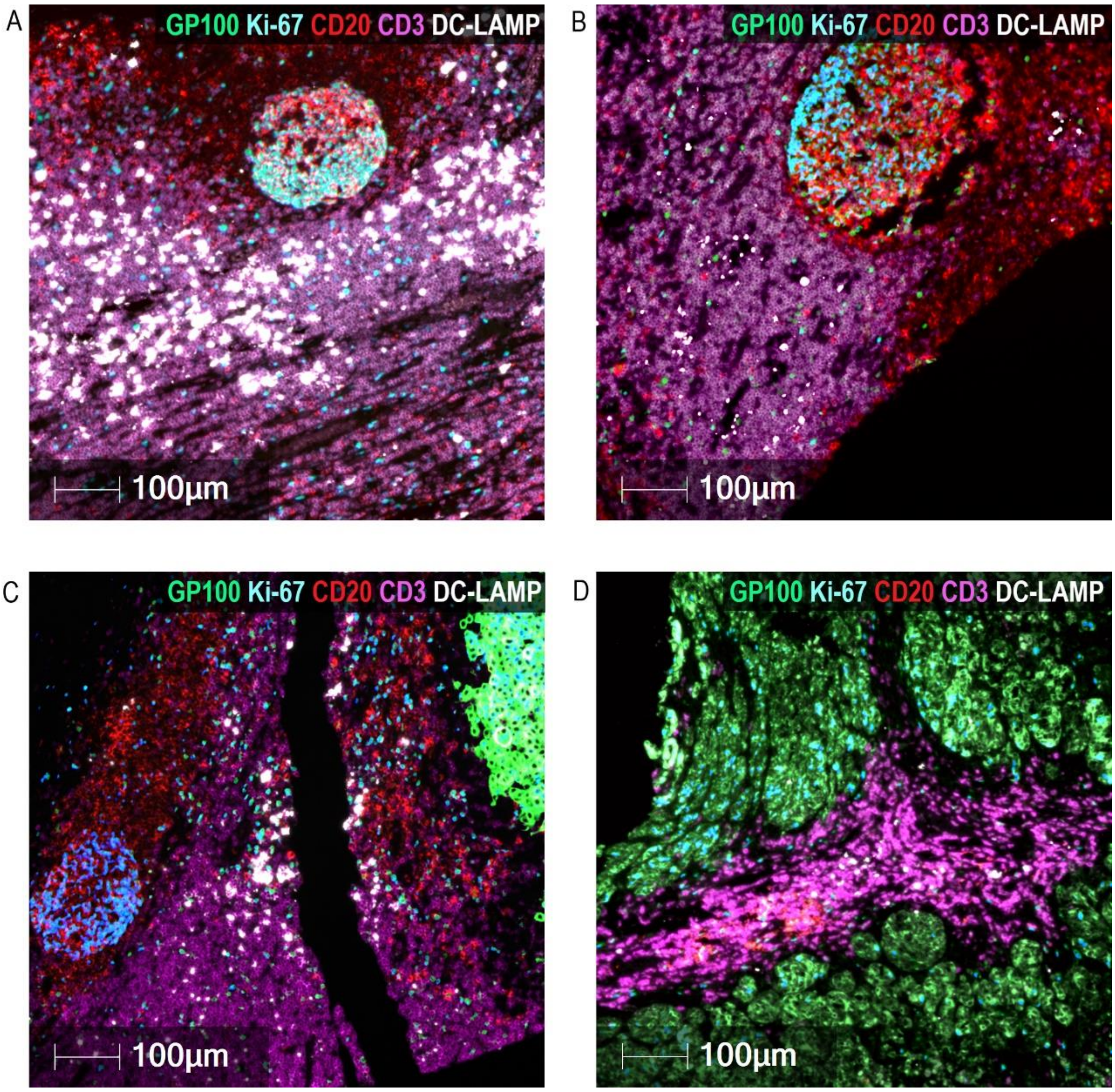

Supplementary Figure 2. Distribution of DC-LAMP$^+$ cells in mTDLN. Multiplex immunofluorescence staining of the mLN peritumoral area of two GO (**A**, patient #2; **C**, patient #17) and two BO (**B**, patient #21; **D**, patient #18) patients with anti-GP100 (green), anti-Ki-67 (blue), anti-CD20 (red), anti-CD3 (purple), and anti-DC-LAMP (white) antibodies. Scale bars: 100µm.
