# Supplementary material for: HEV-associated dendritic cells are observed in metastatic tumor-draining lymph nodes of cutaneous melanoma patients with longer distant metastasis-free survival after adjuvant immunotherapy
Source: Front Immunol. 2023 Aug 25;14:1231734. doi: 10.3389/fimmu.2023.1231734 (PMC10485604; doi:10.3389/fimmu.2023.1231734)
Supplement: Supplementary file 3 [file Image_3.pdf]

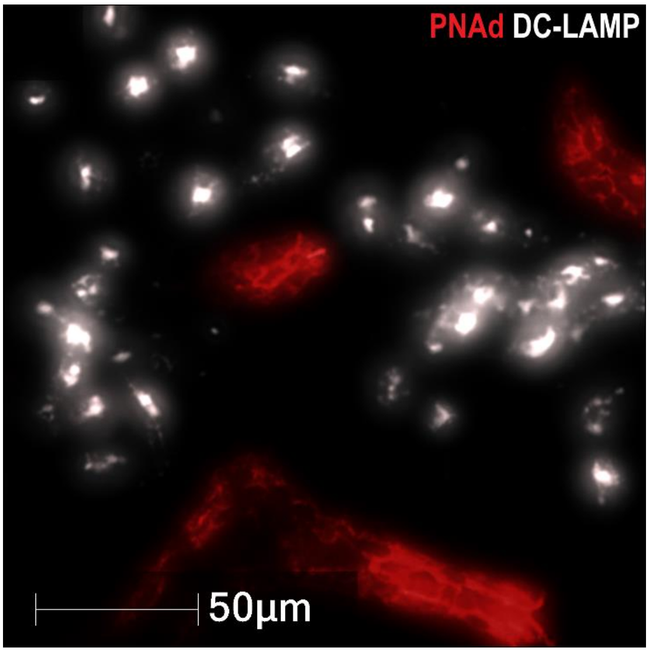

Supplementary Figure 3. **DC-LAMP$^+$ cells surround HEV**. Multiplex immunofluorescence staining of the peritumoral area in mTDLN of one GO (patient #13) with anti-DC-LAMP (white), and anti-PNAd (red) antibodies. Scale Bar: 50µm.
