# Supplementary material for: HEV-associated dendritic cells are observed in metastatic tumor-draining lymph nodes of cutaneous melanoma patients with longer distant metastasis-free survival after adjuvant immunotherapy
Source: Front Immunol. 2023 Aug 25;14:1231734. doi: 10.3389/fimmu.2023.1231734 (PMC10485604; doi:10.3389/fimmu.2023.1231734)
Supplement: Supplementary file 4 [file Image_4.pdf]

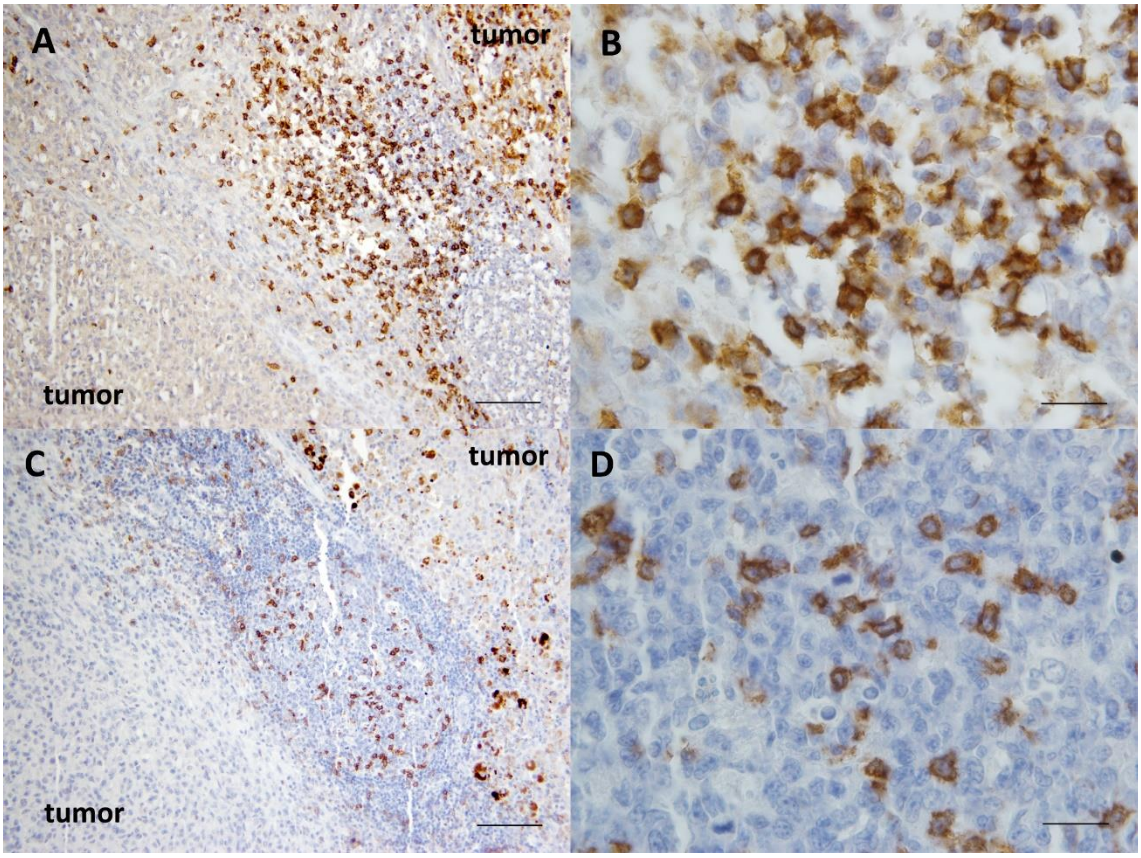

Supplementary Figure 4. CD8$^+$ and PD-1$^+$ cell localization in mTDLN. Representative images evaluating CD8 (A, B) and PD-1 (C, D) expression in mTDLN from GO Patient#17, where PD-1$^+$ cells were fewer than CD8$^+$ cells and were found in the peritumoral area adjacent to the tumor and barely infiltrating the first tumor cell layers. Original magnification: A, C (200X); B, D (1000X). Scale bars: A, C, 100 µm; B, D, 20 µm.
