# Supplementary material for: HEV-associated dendritic cells are observed in metastatic tumor-draining lymph nodes of cutaneous melanoma patients with longer distant metastasis-free survival after adjuvant immunotherapy
Source: Front Immunol. 2023 Aug 25;14:1231734. doi: 10.3389/fimmu.2023.1231734 (PMC10485604; doi:10.3389/fimmu.2023.1231734)
Supplement: Supplementary file 5 [file Image_5.pdf]

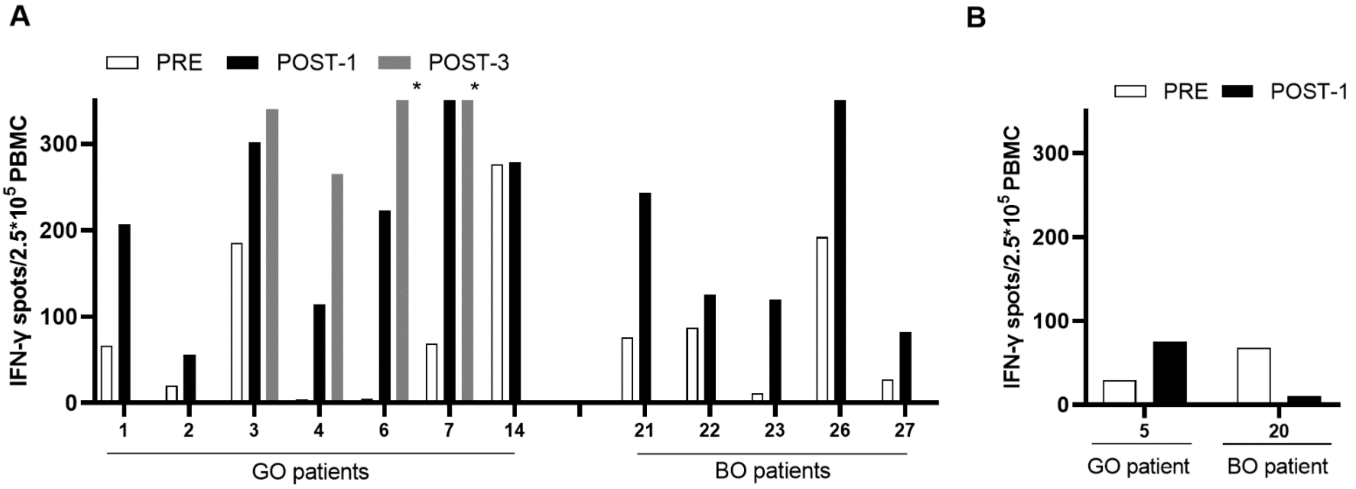

Supplementary Figure 5. IFN-γ enzyme-linked immunospot assay (ELISPOT) analysis in peripheral blood mononuclear cells (PBMC) samples at different time points from GO and BO patients receiving adjuvant immunotherapy. Analysis was performed on available data at PRE (before treatment), POST-1 (6 months after treatment) and POST-3 (24 months after treatment) from (A) vaccinated patients or (B) IFN-α2b treated patients. Each bar corresponds to the mean number of IFN-γ spots quantified for PRE and POST1 PBMC stimulated with the lysate from VACCIMEL as described (*Mordoh J, et al; Front Immunol (2017) 8:625. doi: 10.3389/fimmu.2017.00625*). Spots could be properly quantified from 1 to 350 spots/well/2.5105 PBMC, due to saturation of the signal. More than 350 spots/well were considered TNTC (too-numerous to count).
