# Supplementary material for: HEV-associated dendritic cells are observed in metastatic tumor-draining lymph nodes of cutaneous melanoma patients with longer distant metastasis-free survival after adjuvant immunotherapy
Source: Front Immunol. 2023 Aug 25;14:1231734. doi: 10.3389/fimmu.2023.1231734 (PMC10485604; doi:10.3389/fimmu.2023.1231734)
Supplement: Supplementary file 6 [file Table_1.pdf]

Patient	Age, sex	Primary Tumor			Stage	Treatment	DMFS (months)
		Location	Breslow (mm)	Ulceration			
**1**	37, F	Un	-	-	IIIC	IFN	133 +
**2**	41, F	T	2.3	YES	IIIC	V	141 +
**3**	52, M	L	1.2	NO	IIIB	V	150 +
**4**	47, M	D	3.0	NO	IIIB	V	107 +
**5**	51, M	D	1.2	NO	IIIA	IFN	101 +
**6**	51, F	A	5.5	YES	IIIC	V	97 +
**7**	46, M	T	2.2	YES	IIIC	V	57
**8**	34, M	D	2.0	NO	IIIB	V	54
**9**	29, M	Un	-	-	IIIC	IFN	372 +
**10**	50, F	L	1.5	NO	IIIA	V	80
**11**	17, M	Un	-	-	IIIC	V	360+
**12**	33, F	Un	-	-	IIB	V	112
**13**	44, M	Af	1.65	NO	IIIC	V	348 +
**14**	50, M	D	4.0	NO	IIIB	V	130 +
**15**	40, M	D	0.6	YES	IIIB	V	114+
**16**	56, F	Un	-	-	IIIC	V	213 +
**17**	15, F	A	5.0	YES	IIIC	V	218 +
**18**	42, M	A	5.0	YES	IIIC	V	2
**19**	45, F	T	7.8	YES	IIID	0	2
**20**	41, F	Un	-	-	IIIC	IFN	17
**21**	39, F	L	2.0	YES	IIIB	V	12
**22**	40, M	A	3.2	YES	IIIC	V	10
**23**	35, M	D	4.0	YES	IIIC	V	7
**24**	46, M	D	8.0	NO	IIIC	V	4
**25**	41, F	D	2.9	NO	IIIB	V	6
**26**	52, M	A	8.0	YES	IIIC	V	10
**27**	44, M	L	5.0	YES	IIID	V	10
**28**	53, M	Ah	ND	ND	IIIC	IFN	4
**29**	54, F	T	ND	YES	IIID	IFN	10

Supplementary Table 1. Cutaneous Melanoma patients' characteristics. Patients´ staging was stated as per AJCC 8th edition. #1–17: good outcome (GO) patients, shaded in gray. #18–29: bad outcome (BO) patients. F: female; M: Male. Primary tumor characteristics: A: arm; Af: acral foot; Ah: acral hand; D: dorsal; L: leg;; ND: Not Determined; Un: unknown primary tumor; T: trunk. Treatments after mLN dissection were V: VACCIMEL (22/29); IFN: interferon alpha-2b (6/29); 0: non-treated due to early progression. DMFS: distant metastasis-free survival. (+) censored patients.
